# Association between prognostic nutritional index and the clinical outcomes of patients with acute myocardial infarction: a systematic review and meta-analysis

**DOI:** 10.3389/fcvm.2025.1650043

**Published:** 2025-11-12

**Authors:** Di Wang, Congkang Jia, Tao Yu

**Affiliations:** Department of Cadre’s Ward, The 983rd Hospital of Joint Logistic Support Force of PLA, Tianjin, China

**Keywords:** prognostic nutritional index (PNI), acute myocardial infarction (AMI), prognostic value of survival, meta-analysis, systematic review

## Abstract

**Background:**

Emerging data suggest a correlation between the prognostic nutritional index (PNI) and the clinical outcomes of patients with acute myocardial infarction. Despite these findings, the overall conclusions remain inconclusive.

**Methods:**

A comprehensive literature search was conducted in PubMed, Embase, Web of Science, and the Cochrane Library, covering the period to October 31, 2024, Examining the relationship between PNI and clinical outcomes in AMI patients. The outcomes included mortality due to acute myocardial infarction, major adverse cardiovascular events (MACE), in-hospital mortality, and acute kidney injury. These outcomes were using hazard ratios (HR) and corresponding 95% conﬁdence intervals (CI). Sensitivity and subgroup analysis were used to assess the stability of the results and potential sources of heterogeneity. All analyses were performed using Review Manager 5.4 and STATA 15.0.

**Results:**

Our analysis included twelve retrospective cohort studies and two prospective studies. Demonstrated a correlation between PNI values and mortality (HR = 0.91, 95% CI: 0.91–0.85; *p* < 0.0001). No significant association between the PNI and the incidence of MACE in patients with AMI (HR = 0.85, 95% CI: 0.66–1.11; *p* = 0.23). Sensitivity and subgroup analysis confirmed the stability of the results.

**Conclusion:**

PNI has a strong link to lower mortality rates in patients with AMI, higher the PNI values correspond to lower mortality risk. No significant relationship between PNI and MACE was observed. PNI could be an important factor in individualized managenent of AMI patients in clinical settings.

**Systematic Review Registration:**

PROSPERO, identiﬁer CRD42024620983.

## 1 Introduction

Acute Coronary Syndrome (ACS) refers to a group of clinical syndromes caused by acute myocardial ischemia and hypoxia, typically caused by the rupture or erosion of atherosclerotic plaques and subsequent complete or partial thrombotic occlusion (1). Based on ECG findings and markers of myocardial injury, ACS is classified into Unstable Angina (UA), Non-ST-Segment Elevation Myocardial Infarction (NSTEMI), and ST-Segment Elevation Myocardial Infarction (STEMI). STEMI and NSTEMI, are collectively known as Acute Myocardial Infarction (AMI),—the most severe types of ACS—characterized by high morbidity, mortality, and disability rates (2). In recent years, with an aging population, changing lifestyles, and the prevalence of risk factors, the incidence of AMI has steadily increased, making it a major contributor to global mortality and disability (1). Currently, the primary therapeutic goal in AMI management is to rapidly reopen the infarct-related artery, re-establish adequate blood flow to the heart muscle, preserve endangered myocardial tissue, limit infarct size progression, and maintain cardiac function. Despite the continuous improvement of treatment methods, the mortality rate of AMI is still high, especially the prehospital mortality and reinfarction rate. This underscores the urgent need to enhance early detection, timely treatment and secondary prevention (3).

Acute myocardial infarction (AMI) is one of the most serious diseases of the cardiovascular system, and its morbidity and mortality remain persistently high worldwide. Studies have shown that, beyond traditional risk factors (such as age, hypertension, diabetes, and cardiac function), the nutritional and immune status of patients is increasingly recognized as a key determinant of cardiovascular outcomes (4). In this context, Prognostic Nutritional Index (PNI), an objective marker that evaluates both nutritional and immune function, has gained attention due to its value in predicting clinical outcome. PNI was first proposed by Buzby et al. in 1980 (4, 5). The Prognostic Nutritional Index (PNI), as a simple indicator that integrates serum albumin and lymphocyte counts, has been proven to effectively reflect the body's inflammatory and nutritional balance status, and has shown value in the prognosis prediction of various diseases. However, the predictive efficacy of PNI for all-cause mortality and major adverse cardiovascular events (MACE) in patients with ACS is inconsistent among existing studies (6). In a study by between prognostic nutritional indices and five-year survival outcomes in individuals who underwent emergency coronary artery bypass grafting due to acute ST-elevation myocardial infarction, tracking a cohort of 131 patients over the study period. The authors found that PNI was significantly associated with long-term mortality in individuals diagnosed with AMI (7).

Although numerous clinical studies have explored the relationship between the PNI and the prognosis of AMI patients, the conclusions remain inconsistent. These discrepancies may be attributed to variations in sample sizes, geographic regions, threshold definitions, and other methodological differences (8). Therefore, the purpose of this paper is to synthesize existing clinical evidence through a systematic review and meta-analysis, in order to derive a more robust and comprehensive conclusion. This willhelp to determine whether PNI can effectively predict the prognosis of AMI patients, so as to provide the latest theoretical basis for clinical construction of accurate prediction models.

## 2 Materials and methods

### 2.1 Literature search

In order to provide a transparent account of the methods used, this study adhered to the guidelines outlined in the PRISMA 2020 (9), and its protocol was registered in the International Prospective Systematic Evaluation Registry (PROSPERO: CRD42024620983). Two researchers (WD and JCK) independently formulated the database search plan. They selected relevant subject headings and keywords to systematically search PubMed, Embase, Web of Science, and the Cochrane Library for studies published up to October 31, 2024. The search strategy incorporated a broad spectrum of terms, including: “Myocardial Infarction,” “Infarction, Myocardial”, “Infarctions, Myocardial”, “MyocardialInfarctions”, “Heart Attack”, “Heart Attacks”, “Myocardial Infarct”, “Infarct, Myocardial”, “Infarcts, Myocardial”, “Myocardial Infarcts”, “Cardiovascular Stroke”, “Cardiovascular Strokes”, “Stroke, Cardiovascular”, “Strokes, Cardiovascular” and “prognostic nutritional index”, “PNI”. Supplementary Table S1 provides the complete search methodology employed in this review.

### 2.2 Study selection

Eligible studies had to fulfill the following conditions: (1) the participants had a confirmed diagnosis of acute myocardial infarction by standard clinical criteria; (2) the prognostic nutritional index (PNI) was reported, calculated as albumin (g/L) + 5 × lymphocyte count (10^9^/L), and patients were grouped based on whether their PNI was above or below 45; (3) examined how PNI related to outcomes in AMI patients; (4) the study presented or allowed calculation of HRs or ORs with 95% confidence intervals; (5) a clear cut-off was used to divide high-PNI and low-PNI groups; (6) the study had to be fully published in a peer-reviewed journal.

The following exclusion criteria were applied: (1) articles such as reviews, editorials, conference summaries, letters, and individual case reports were not included; (2) studies without adequate data to calculate HR or OR and 95% CI were excluded; (3) studies that did not include survival data were excluded; (4) studies with duplicate or overlapping data were also removed. Two researchers (WD and JCK) independently reviewed the titles and abstracts of studies retrieved from databases, downloaded full-text articles, and assessed them to collect eligible studies. Any disagreements in study selection were addressed through discussion until a mutual agreement was reached.

### 2.3 Data extraction

Two researchers (WD and JCK) independently performed data extraction. Any discrepancies were resolved through consensus involving all co-authors. Information extracted consisted of: the first author's surname, year of publication, date extraction of publication, country, study design, period of study, number of participants, numbers of cases and controls; Mean/median Age; PNI cut-off;Population; Detection timing; treatment; HR/OR and 95% confidence interval (CI) indicating the highest acute myocardial infarction mortality, MACE, in hospital mortality and acute kidney injury. It is important to note that, for studies providing PNI data, the inverse of the reported HR or OR values and their confidence intervals was calculated, with the upper and lower limits reversed accordingly, to standardize the data for statistical analysis.

### 2.4 Quality assessment

We assessed the quality of the analyzed publications with the Newcastle-Ottawa Scale (NOS), rating them on selection, comparability, and outcome criteria. Studies achieving a score between 6 and 9 were deemed to be of high quality.

### 2.5 Statistical analysis

The pooled HRs or ORs with associated 95% CIs were calculated to determine the prognostic value of PNI in individuals diagnosed with acute myocardial infarction to indicating the highest acute myocardial infarction mortality, MACE, in hospital mortality and acute kidney injury. We used Cochran's *Q* and Higgins' *I*^2^ to evaluate heterogeneity, considering it significant when *P* < 0.05 or *I*^2^ > 50%. Random-effects model was employed for all data analysis. Subgroup and sensitivity analyses were performed to confirm the stability of results for OS and PFS. The source of heterogeneity was also discussed. Funnel plots alongside Egger's regression test were utilized to evaluate potential publication bias. All analyses were performed using STATA version 15.0 and Review Manager version 5.4.

## 3 Results

### 3.1 Study characteristics

**Figure 1:**
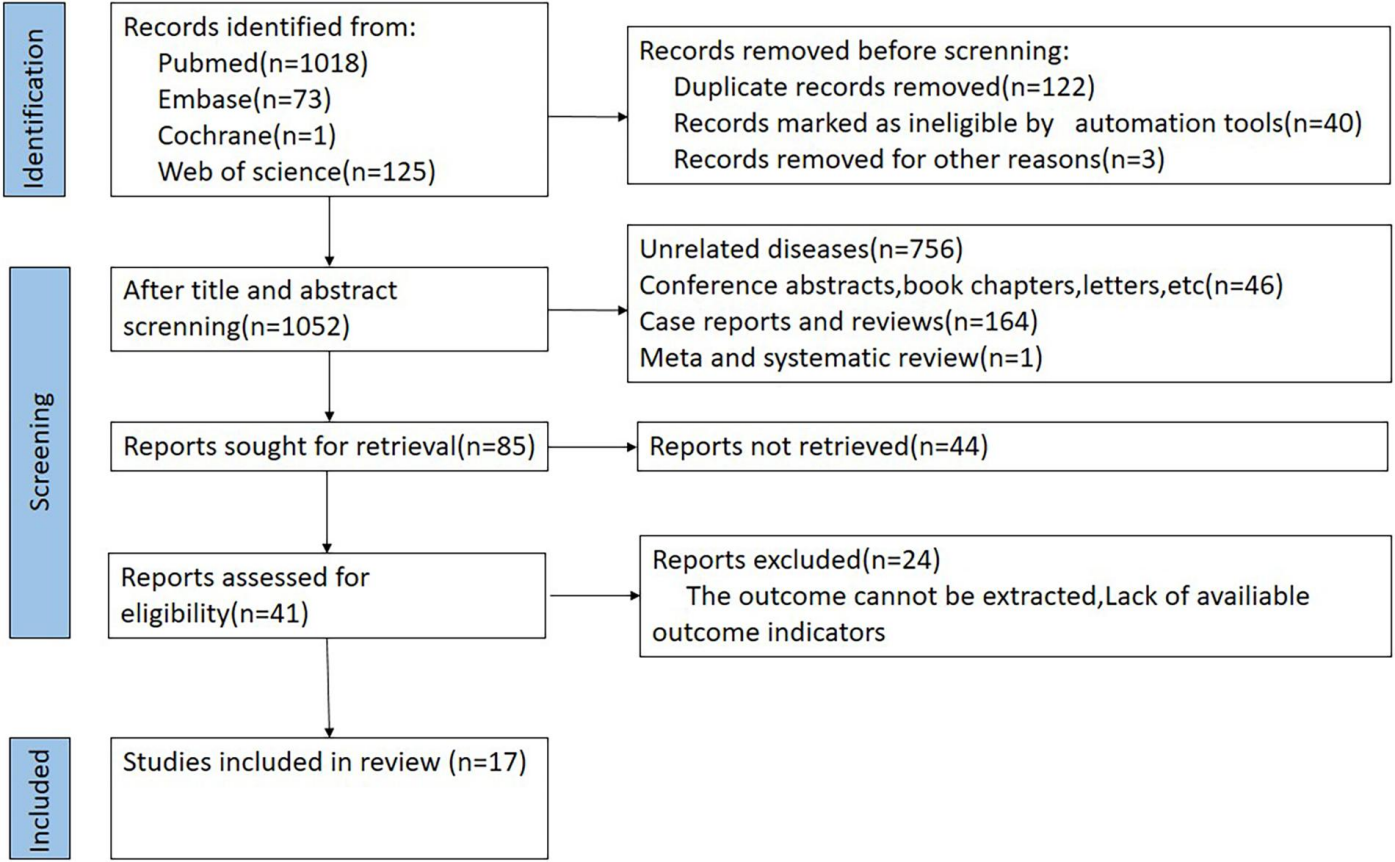
Flow chart of literature screening.

From an initial pool of 1,217 records, 122 duplicates were eliminated. Screening titles and abstracts led to the exclusion of 1,052 articles. Following full-text review of the remaining studies, 26 were excluded for lacking sufficient survival data. Seventeen eligible studies, covering 42,146 patients in total, were finally included in this meta-analysis (Figure 1) (7, 9–24).

Among the seventeen included studies, nine were from Europe and eight from Asia. Among them incorporated twelve retrospective cohort studies (7, 9–12, 14–16, 18–24), whereas the remaining two were prospective (13, 17). All cohort studies, published in English between 2017 and 2024, investigated the prognostic value of PNI by comparing outcomes between high-PNI and low-PNI groups. In terms of PNI measurement, all studies measured PNI at admission as baseline, three studies measured PNI at admission and after or before prospertive, four evaluated PNI during preoperative, intraoperative, and postoperative. Regarding the assessment of PNI, thirteen articles explored its potential to predict mortality, while eight others investigated its prognostic relevance MACE outcomes. Table 1 presents key features of the studies selected for analysis.

**Table 1:** Basic characteristics of the included literature.

Author mean + range; mean + SD; mean + SE; median + range; median + IQR	Study period	Region	Study design	Population	Testing timing	Treatment methods	No. of patients	Gender		Mean/median Age	PNI cut-off	Quality Score
								Male	Female			
Basta et al. (10)	2006.01–2012.12	Pisa, Italy.	Prospective cohort	STEMI with primary PCI	At admission	PCI + hospital medications	945	705	240	65.7 ± 12.5	35–38	7
Chen et al. (9)	2011.09–2014.11	Xinjiang, China	Prospective cohort	STEMI within 12 h of undergoing pPCI	At admission, after pPCI	PCI + hospital medications	309	250	59	58.3		7
Chen et al. (11)	2016.10–2017.09	Sichuan, China	Prospective cohort	diagnosed with AMI	At admission	NA	598	456	142	64 ± 13		6
Demirci et al. (7)	2013–2018	Istanbul, Türkiye	Retrospective cohort	did not qualify for pPCI and required emergent CABG	Preoperative, intraoperative, and postoperative	CABG	131	111	20	57.0 ± 10.6.	44.9	8
Kekin et al. (14)	2011.07–2012.12	Italian	Retrospective cohort	STEMI with primary PCI	At admission	PPCI + Out-hospital medication	1,823	1,493	330		44	8
Kim et al. (15)	2013.01–2015.12	Republic of Korea	Retrospective cohort	AMI	During admission	Hospitalization	1,147	832	315	65.6	50	6
Li et al. (17)	2017.09–2020.04	Shanghai, China	Retrospective cohort	elderly AMI patients in CCU	At admission	NA	307	208	99	75.28 ± 9.44	40.923	6
Sun et al. (20) (L-PNI/H-HbA1c)	2021.09–2022.01	Beijing, China	Retrospective cohort	T2DM with ACS underwent PCI	At admission	PCI + hospital medications	2,005	1,426	579	60.77		
L-PNI/L-HbA1c												
Wang et al. (21)	2016.01.01–2019.03.31	Guangdong, China	Retrospective cohort	elderly AMI patients in CCU	At admission	Hospitalization	273	170	103	81.2 ± 4.2		6
Xie et al. (22)	2016.02–2022.01	China	Retrospective cohort	NOAF with STEMI underwent PCI	24 h of symptom onset	Hospitalization	600	484	116	63.61	40.1	6
Yildirim et al. (23)	2014.01–2015.01	Turkey	Retrospective cohort	NSTEMI with PCI	At admission	Hospitalization	915	471	444	73.1 ± 9.0		6
Hatem et al. (12)	2019.01–2019.06	Turkey	case-control cohort	CA-AKI in NSTEMI patients undergoing CAG.	At admission and 48 h after the procedure.	Treated for NSTEMI	336	245	91	62.0 ± 12.7	48.50%	6
Huang et al. (13)	2008–2019	Israel	Retrospective cohort	AMI (≥ 18 years) patients in ICU	At admission	Hospitalization	1,180	781	399	68	41.5	6
Kurtul et al. (16)	2017.01–2020.02	Turkey	case-control cohort	STEMI with pPCI	After admission and before coronary angiography procedures, and thereafter once a day throughout hospitalization	PPCI	836	635	201	58 ± 12	38	7
Ling et al. (18)	2007.01–2020.12	China	Retrospective cohort	AMI and a history of PCI	At admission or before PCI		17,661				45.1	6
median: 42.7 < PNI ≤ 48.2							5,895	4,803	1,092	61.5 ± 11.6		
low: PNI ≤ 42.7							5,856	4,992	864	65.7 ± 11.4		
Safak et al. (19)	2016.01–2018.12	Turkey	Retrospective cohort	STEMI and underwent pPCI	At admission	PPCI + hospital medications	404	145	259		37	7
No-reflow (+)										62.1 ± 7.9		
Non-reflow (–)										60.6 ± 8.1		
Yukel and Kose (24)	2016.08–2020.12	Turkey	Retrospective cohort	ACS, older than 18 years undergoing CAG	On admission and after the procedure and follow-up 3–5 days after discharge		925	680	245	62.5 ± 12.4	48.6	6
CIN negative										60.3 ± 11.8		
CIN positive										68.9 ± 12.2		

### 3.2 Study quality

All included studies received NOS scores between 6 and 8, reflecting a high level of study quality (refer to Supplementary Table S2).

### 3.3 Meta-analysis results

#### 3.3.1 PNI and mortality

**Figure 2:**
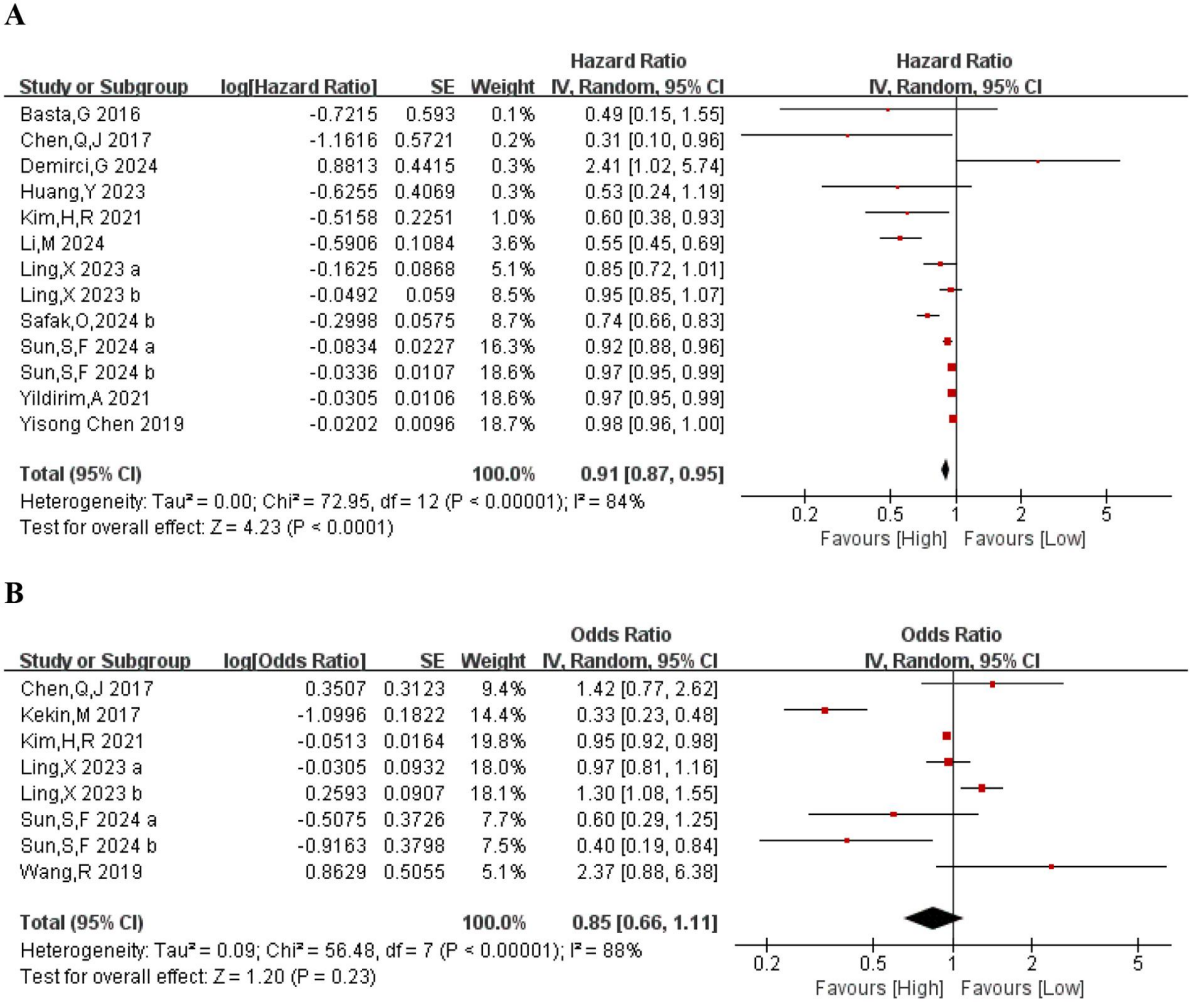
**(A)** Forest plots for the association between PNI and mortalitty; **(B)** forest plots for the association between PNI and MACCE.

We evaluated the association between PNI and mortality across fifteen cohort studies, encompassing two outcome indicators and a total of 42,146 participants. Each of these studies, all reported PNI values at admission. Four studies offered data from before and after admission, while two provided preoperative, intraoperative, and postoperative PNI values. Due to substantial heterogeneity across indcluded studies (*I*^2^ = 87%, *p* < 0.0001), a random-effects model was applied (Figure 2A). The results demonstrated a strong and positive association between the PNI and mortality (HR = 0.91, 95% CI: 0.91–0.85; *p* < 0.0001, Figure 2A).

Table 2 presents the results of these analyses. Mortality data were stratified by study design, sample size, region, mean age, and PNI cut- off. PNI showed no prognostic value in prospective studies, European populations, or in studies using a cut-off value ≥40. In contrast, retrospective studies demonstrated a significant link with mortality (HR = 0.88, 95% CI: 0.83–0.84; *p* < 0.0001). Additionally, subgroup analysis in Asian populations revealed a signiﬁcant predictive value (HR = 0.91, 95% CI: 0.87–0.96; *p* = 0.0009). Finally, the subgroup analysis using a PNI cut-off <40 also showed a significant predictive value (HR = 0.94, 95% CI: 0.94–0.91; *P* = 0.0003).

**Table 2:** Pooled HRs for mortality and MACE in subgroup analyses.

Subgroup	Mortaltiy				MACE			
	Study	HR [95%CI]	*P* va1ue	*I^2^*	Study	OR [95%CI]	*P* va1ue	*I^2^*
Total	13	0.91 [0.87–0.95]	<0.0001	84%	8	0.85 [0.66–1.11]	0.23	88%
Study design								
Prospective	3	0.63 [0.30–1.34]	0.23	63%	1	1.42 [0.77–2.62]	0.26	NA
Retrospective	10	0.88 [0.83–0.94]	<0.0001	86%	7	0.81 [0.62–1.06]	0.13	89%
Sample size								
≥1,000	5	0.93 [0.88–0.98]	0.009	59%	5	0.67 [0.40–1.10]	0.11	92%
<1,000	8	0.87 [0.80–0.94]	0.0007	90%	3	1.23 [0.77–1.97]	0.38	59%
Region								
Asia	8	0.91 [0.87–0.96]	0.0009	84%	7	1.02 [0.83–1.24]	0.88	74%
Europe	6	0.86 [0.67–1.11]	0.26	86%	1	0.33 [0.23–0.48]	<0.0000	NA
Mean/median age								
≥65 years	6	0.76 [0.62–0.93]	0.009	85%	3	1.17 [0:86–1.60]	0.33	86%
<65 years	8	0.92[0.87–0.97]	0.002	84%	5	0.65[0.36–1.16]	0.14	88%
PNI cut-off								
≥40	4	0.72 [0.45–1.16]	0.18	72%	3	1.05 [0.87–1.26]	0.61	82%
<40	9	0.94 [0.91–0.97]	0.0003	78%	5	0.73 [0.35–0.51]	0.4	85%

HR, hazard ratio; CI, conﬁdence interval; NA, not available; MACE, major adverse cardiovascular events.

#### 3.3.2 PNI and MACE

Of the seventeen included articles, eight provided the necessary data on major adverse cardiovascular events (MACE), encompassing a total of 23,218 participants (9, 15, 16, 18, 21, 22). The analysis showed no notable relationship between the PNI and the incidence of MACE in individuals diagnosed with AMI (HR = 0.85, 95% CI: 0.66–1.11; *p* = 0.23). Subgroup analyses showed no consistent predictive value, except in the European subgroup (HR = 0.33, 95% CI: 0.23–0.48; *p* = 0.0003), though this result is derived from one single study and should therefore be approached with caution. Regional differences in the occurrence and reporting of myocardial infarction, as well as age-related variations in nutritional status, may partly explain the heterogeneity in the prognostic performance of PNI across populations.

### 3.4 Sensitivity analysis

**Figure 3:**
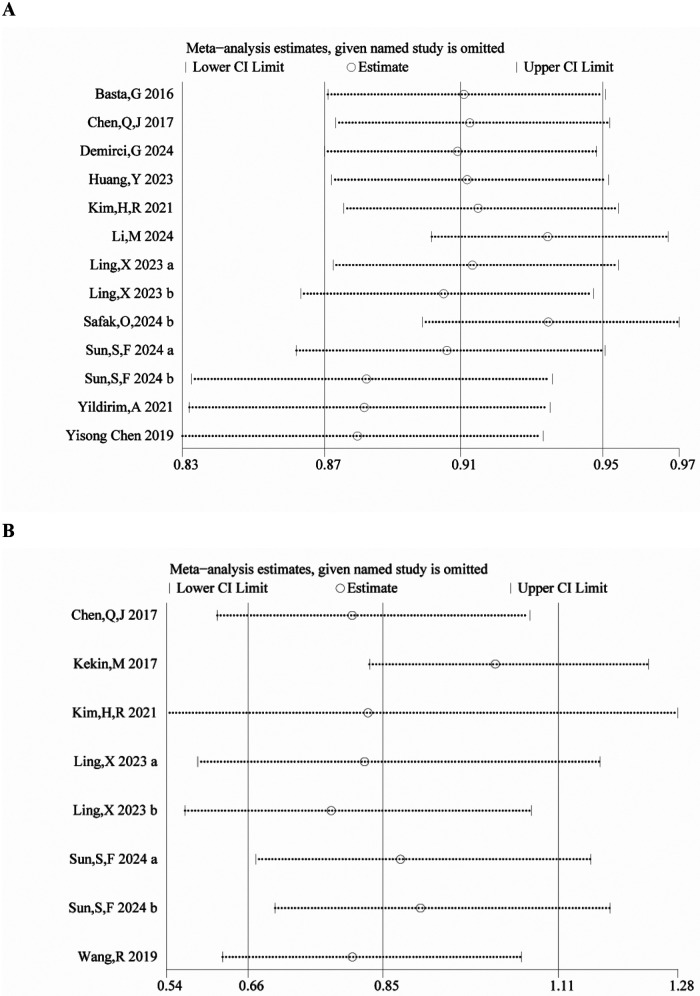
Sensitivity analysis of **(A)** mortality and **(B)** MACCE.

We performed a sensitivity analysis to assess the robustness of our findings regarding the clinical significance of PNI. The pooled effect size remained stable after sequential exclusion of each individual study, indicating that no individual study had an outsized impact on the overall outcomes for either mortality (Figure 3A) or MACE (Figure 3B). These findings support the reliability of our conclusions.

**Figure 4:**
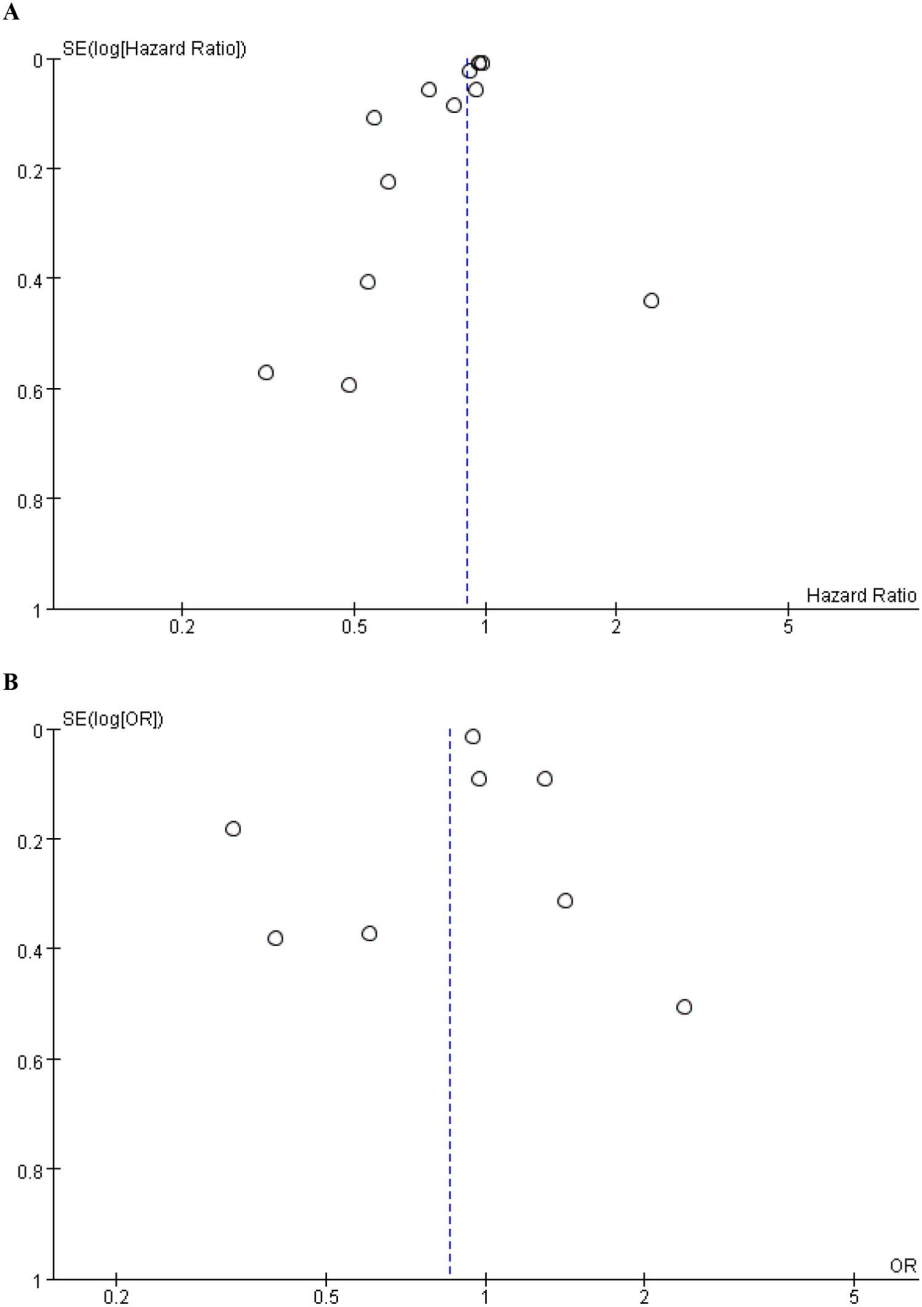
Funnel plot for the evaluation of publication bias for **(A)** mortality and **(B)** MACCE.

### 3.5 Publication bias

Publication bias was evaluated through a funnel plot and Egger's test. Visual inspection of the funnel plot suggested the presence of publication bias for mortality outcomes (Figure 4), but not for MACE. This was further supported by Egger's test, which revealed a significant publication bias in studies reporting mortality (*P* = 0.016), whereas no significant bias was detected for MACE (*P* = 0.721).

## 4 Discussion

Acute myocardial infarction (AMI) is one of the most serious clinical events in cardiovascular disease. Its pathophysiological mechanisms involve rupture of atherosclerotic plaques, thrombosis, and myocardial ischemic necrosis. Recent studies have found that inflammatory response and platelet activation are crutial in the occurrence and development of AMI (6, 25–27). The PNI is a novel biomarker that reflects the dynamic level of platelet and neutrophil aggregation, which is at the core of the thrombosis and inflammation cascade. It may be an important index to evaluate the risk and prognosis of thrombosis in AMI patients. Research has indicated that PNI levels in peripheral blood of AMI patients in acute stage are significantly increased, suggesting that the formation of platelet-neutrophil complexes may further aggravate myocardial injury by promoting thrombus stability, worsening vascular endothelial damage, and inflammatory response (21, 26). However, studies on the relationship between PNI and short-term and long-term prognosis of AMI patients are still limited, and its specific mechanisms and clinical application value require more evidence-based evidence-based medical support. Further study on the pathophysiological significance of PNI in AMI may provide new ideas for optimizing risk stratification and individualized antithrombotic therapy. Therefore, the aim of this paper was to explore the relationship between PNI and the prognosis of AMI patients through systematic review and meta-analysis, in order to obtain the latest comprehensive evidence in support of evidence-based medicine. It was found that PNI could predict mortality, but not MACE. Our sensitivity analysis confirmed that both indicators were stable and reliable. Moreover, publication bias analysis found that there was a publication bias in mortality, which we speculate may be related to the differences in sample size, publication region and other factors. Therefore, more large-scale, multi-center studies should be conducted to further address the impact of publication bias, so as to further confirm the findings of this paper. In addition, we found that Zhang et al. (28) published a meta-analysis in 2023 that assessed if prognostic nutritional index (PNI) can predict mortality and major adverse cardiac events (MACE) in coronary artery disease (CAD) patients. The meta-analysis revealed that PNI is a key predictor of mortality in CAD patients, with individuals having a low PNI showing a notably higher incidence of MACE (28). Therefore, we speculate that PNI may not only be used to predict outcomes in AMI, but may also have similar predictive value in other cardiovascular diseases. This further supports the notion that PNI can be used as a general prognostic marker across the spectrum of cardiovascular conditions.

In the subgroup analysis, we focused on regions and threshold values. We found that in Asia, PNI was valuable for predicting mortality but not for MACE, whereas in Europe, it was not valuable for predicting mortality but showed predictive value for MACE. The predictive value of PNI varies by region. In Asian populations, PNI has predictive value for all-cause mortality but not significantly for MACE; in contrast, the situation is reversed in European populations. This heterogeneity may be caused by multiple factors. Firstly, there are differences between Asian and European populations in terms of genetic background, dietary structure, average body mass index, and the spectrum of comorbidities, which may affect the strength of the association between nutritional status and different endpoint events. Secondly, differences in medical practice between the two regions, such as revascularization strategies, drug prescription patterns, and patient follow-up management systems, may have altered the occurrence patterns of death and MACE events, thereby modulating the predictive performance of PNI. According to the latest guidelines, regional population characteristics also affect the results, as Asians are more numerous and have a higher incidence of disease than Europeans, making the results more pronounced. From the analysis of threshold values, it was concluded that when PNI ≥ 40, its predictive value is not as strong as when PNI < 40. Therefore, in future research model construction, it may be more effective to set the PNI cut-off value below 40 to maximize its predictive potential.

The occurrence and prognosis of acute myocardial infarction (AMI) are influenced by many factors, among which nutritional status and immune-inflammatory response have been proved to be important regulatory factors in recent years (27, 29). The initial inflammatory response after AMI helps clear dead tissue, but persistent inflammation can lead to myocardial fibrosis and ventricular remodeling. Low PNI (low albumin + low lymphocyte) reflects the following pathological processes. Hypoalbuminemia weakens the neutralization of pro-inflammatory factors such as IL-6 and CRP, and aggravates systemic inflammation (30, 31). Lymphocytopenia (especially Th1/Th2 imbalance) leads to dysregulation of inflammation and promotes plaque instability and thrombosis. In chronic inflammatory states, cytokines (such as TNF-α) promote muscle proteolysis and lipolysis via the NF-κB pathway, leading to cachexia. Inflammatory factors damage the intestinal epithelium, increase permeability, trigger endotoxemia, further inhibit albumin synthesis and exacerbate inflammation (32). Albumin is a key protein in maintaining colloid osmotic pressure and transporting nutrients such as fatty acids and hormones. Hypoalbuminemia leads to tissue edema caused by decreased plasma osmotic pressure, which aggravates myocardial ischemia. The decrease of albumin leads to the decrease of free radical scavenging ability and accelerates the apoptosis of cardiomyocytes. The high metabolic state after AMI increases the energy demand. If the nutrient intake is insufficient, the body breaks down muscle protein for energy and weakens the myocardial repair ability (30). PNI fully reflects the patient's immune-nutrition-inflammation axis by integrating albumin (nutritional/inflammatory marker) and lymphocytes (immune marker). Therefore, the conclusion of our paper suggests that PNI can predict the prognosis of AMI patients is biologically reasonable.

Our meta-analysis, though informative, has some limitations. Most of the studies included were from Asia, specifically China. Therefore, the conclusions should be viewed in the context of this geographical focus, and caution is needed when generalizing the results to populations in Europe, Africa, the Americas, or elsewhere. Additional studies are required to verify the prognostic relevance of PNI for AMI patients in non-Asian populations. Although the majority of included studies have adjusted for the association between the Prognostic Nutritional Index (PNI) and acute myocardial infarction (AMI) prognosis through multivariate analysis—typically incorporating covariates such as age, sex, diabetes, hypertension, and smoking history to control for the influence of these conventional cardiovascular risk factors—it must be noted that variations exist across studies in terms of variable selection and adjustment scope. Some studies may not have included all relevant clinical characteristics or sociodemographic variables. Furthermore, potential confounding factors, including diabetes, hypertension, age, and sex, were not systematically evaluated or adjusted for in all studies. Consequently, while existing evidence suggests that PNI may possess predictive value for AMI prognosis, the strength and reliability of its independent association require further validation under more comprehensive and consistent control conditions due to potential residual confounding. Egger's test indicated the presence of publication bias in the studies on all-cause mortality (*p* < 0.05). This bias might stem from small sample studies or the non-publication of negative results. To assess the impact of this bias on the reliability of the conclusion, The results showed that after filling in the potential missing studies, the estimated combined hazard ratio (HR) did not change substantially, and the association remained statistically significant. This suggests that despite the publication bias, the main conclusion of this study regarding the correlation between PNI and all-cause mortality is robust. Secondly, There was high heterogeneity in individual studies and instability in sensitive analyses, Our results may be less reliable at this time, and assumptions about uncertainty in the study evaluation data and methods of use may affect the robustness of the combined results.

## 5 Conclusion

Our study found that PNI can significantly predict mortality in patients with AMI, and the higher the PNI index, the lower the mortality rate. However, our study found no significant correlation between PNI and MACE in patients with AMI. In addition, our research suggests that the predictive value of PNI is greater in Asian populations than in European ones. Considering certain limitations of this paper, such as the small sample size and potential publication bias, more international, multicenter, prospective clinical studies are needed to further confirm the predictive value of PNI in AMI patients and to identify possible influencing factors.

## Data Availability

The original contributions presented in the study are included in the article/Supplementary Material, further inquiries can be directed to the corresponding author's.
