# Supplementary material for: Association between prognostic nutritional index and the clinical outcomes of patients with acute myocardial infarction: a systematic review and meta-analysis
Source: Front Cardiovasc Med. 2025 Nov 12;12:1650043. doi: 10.3389/fcvm.2025.1650043 (PMC12647068; doi:10.3389/fcvm.2025.1650043)
Supplement: Supplementary file 1 [file Table1.docx]

TableS1 Literature Search Strategy

PubMed-1018

**(("Myocardial Infarction"[Mesh]) OR (((((((((((((Infarction, Myocardial) OR (Infarctions, Myocardial)) OR (Myocardial Infarctions)) OR (Heart Attack)) OR (Heart Attacks)) OR (Myocardial Infarct)) OR (Infarct, Myocardial)) OR (Infarcts, Myocardial)) OR (Myocardial Infarcts)) OR (Cardiovascular Stroke)) OR (Cardiovascular Strokes)) OR (Stroke, Cardiovascular)) OR (Strokes, Cardiovascular))) AND ((prognostic nutritional index) OR (PNI))**

Embase-73


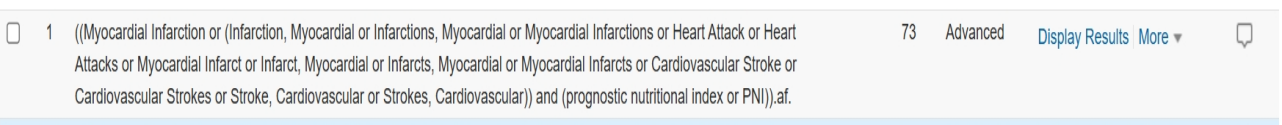


Cochrane-1


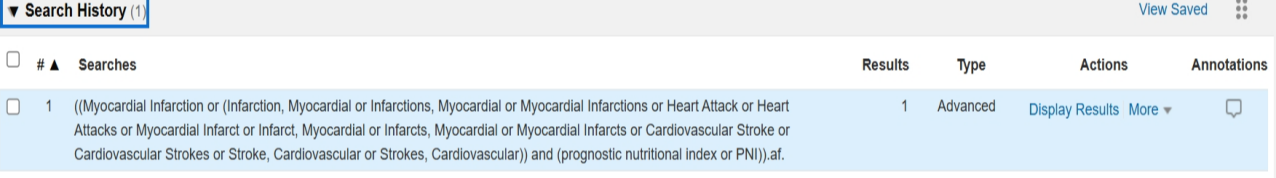


Web of science-125

**((Myocardial Infarction) OR (((((((((((((Infarction, Myocardial) OR (Infarctions, Myocardial)) OR (Myocardial Infarctions)) OR (Heart Attack)) OR (Heart Attacks)) OR (Myocardial Infarct)) OR (Infarct, Myocardial)) OR (Infarcts, Myocardial)) OR (Myocardial Infarcts)) OR (Cardiovascular Stroke)) OR (Cardiovascular Strokes)) OR (Stroke, Cardiovascular)) OR (Strokes, Cardiovascular))) AND ((prognostic nutritional index) OR (PNI))** (Topic)
